# Antifungal Susceptibility of *Candida albicans* Isolated from Tongue and Subgingival Biofilm of Periodontitis Patients

**DOI:** 10.3390/antibiotics11060802

**Published:** 2022-06-14

**Authors:** Milena Radunovic, Milena Barac, Jovana Kuzmanovic Pficer, Dusan Pavlica, Aleksandar Jovanovic, Ana Pucar, Sanja Petrovic

**Affiliations:** 1 Department of Microbiology and Immunology, School of Dental Medicine, University of Belgrade, Dr Subotica 1, 11000 Belgrade, Serbia; dusan.pavlica@stomf.bg.ac.rs; 2 Department of Oral Medicine and Periodontology, School of Dental Medicine, University of Belgrade, Dr Subotica 4, 11000 Belgrade, Serbia; milena.barac@stomf.bg.ac.rs (M.B.); ana.pucar@stomf.bg.ac.rs (A.P.); 3 Department for Medical Statistics and Informatics, School of Dental Medicine, University of Belgrade, Dr Subotica 1, 11000 Belgrade, Serbia; jovana.kuzmanovic@stomf.bg.ac.rs; 4 Clinic of Urology, University Clinical Center of Serbia, Resavska 51, 11000 Belgrade, Serbia; sasaj11385@gmail.com

**Keywords:** *Candida*, antifungal agents, drug resistance, antifungal, periodontitis

## Abstract

The subgingival biofilm, as the most complex microbial community, has been proven to be reservoir of *Candida* spp. The main concept of this study was to investigate if there is a difference between the sensitivity of *Candida albicans* (*C. albicans*) isolated from tongue and subgingival areas of periodontitis patients to antifungal agents. The aim of the study was to determine: (1) the distribution of different *Candida* species in the tongue and subgingival samples of periodontitis patients; (2) the susceptibility of *Candida albicans* strains from tongue and subgingival biofilm to the effects of commonly used antifungal agents: fluconazole, amphotericin B and itraconazole; (3) the correlation between the susceptibility of *Candida albicans* and clinical periodontal parameters. Tongue and subgingival biofilm samples of periodontitis subjects (N = 163) were examined. Susceptibility was tested when the same *Candida* species was isolated from both sites (17 subjects). *Candida* spp. were isolated in 23.3% of tongue and 21.5% of the subgingival samples. All isolates were susceptible to amphotericin B, while 64.71% of tongue and 52.94% of subgingival isolates were susceptible to fluconazole. A low frequency of itraconazole susceptibility was observed for tongue (17.64%) and subgingival isolates (11.76%). The correlations between full-mouth plaque score and Minimal Inhibitory Concentration (MIC) for tongue isolates were strongly positive for all antimycotics. Positive correlation was also observed between moderate periodontal destruction and MICs for tongue and subgingival isolates. The susceptibility of *C. albicans* to antifungals correlate with oral hygiene and moderate periodontal destruction. There is no difference in antifungal susceptibility between tongue and subgingival isolates.

## 1. Introduction

The oral cavity provides multiple niches for various microbes, including yeasts. The most frequently isolated yeast that inhabits the oral cavity is *Candida* spp. *Candida* spp. is most commonly isolated from the tongue dorsal surface, followed by the palate and buccal mucosa [1]. Lately, there have been findings of *Candida* spp. in subgingival areas [2] and pulp systems [3,4]; these areas have been less explored in this relation. 

The most frequently isolated *Candida* species from the oral cavity is highly adaptable *Candida albicans* (*C. albicans*), but other non-albicans species have also been found [5]. In healthy individuals, *Candida* spp. is part of the oral microbiome [6], which maintains homeostasis of the oral cavity. A shift in the oral microbiome leads to dysbiosis, which may further affect the metabolism and virulence of microorganisms [7]. 

Periodontitis, an inflammatory and destructive disease of the tooth supporting tissues, is regarded as a state of local dysbiosis [8]. Periodontal pockets, as a pathognomonic sign of periodontitis, contain stable dysbiotic complex biofilm masses attached to non-shedding tooth surfaces [9]. This provides optimal conditions for a variety of microorganisms [2]. As mentioned, *Candida* spp. is among them [2,10]. Due to the fact that the epithelium of periodontal pockets is inflamed and ulcerated, microorganisms from periodontal pockets may easily enter the bloodstream [11]. In immunocompromised patients, the spreading of microorganisms from the oral mucosa and periodontal pockets can lead to a severe systemic infection with a high mortality rate [12], such as cardiovascular diseases including endocarditis, diabetes bacterial pneumonia, pre-term birth and low birth weight [13,14,15,16]. Lately, there has even been growing concern about the potential role of yeasts originating from the oral cavity in the etiopathogenesis of fungal bacterial endocarditis [17]. 

Considering the information mentioned above as well as the fact that microorganisms grown in biofilms are more resistant (up to 250-fold) than planktonic forms [18,19], we decided to explore not only the frequency but also the susceptibility of yeasts residing in subgingival biofilms. 

It has already been proven that the resistance determinants of bacterial isolates from periodontal pockets are more prevalent and abundant than those of isolates from gingival pockets (where only gingivitis is present—a reversible inflammation without destruction) [20]. The fact that the biofilm of periodontal pockets is more complex than the biofilm of gingival pockets may reveal the importance of biofilm complexity regarding microorganism resistance. Such investigation was not done for yeasts. We assumed that *Candida* spp. isolated from subgingival areas of periodontal pockets could be more resistant to antimycotics than *Candida* spp. isolated from a less complex and ecologically different biofilm of the tongue. 

The main concept of this study was to investigate if there is a difference between the susceptibility of *Candida albicans* isolated from the tongue and subgingival areas of periodontitis subjects to the effects of three commonly used antifungal agents. In order to achieve this, three main aims were defined: (1) analysis of the distribution of different *Candida* spp. on the samples recovered from the tongue and periodontal pockets of patients affected by periodontitis; (2) evaluation of difference in the susceptibility of *C. albicans* strains isolated from the tongue and subgingival areas of patients with periodontitis to the commonly used antifungal agents: fluconazole, amphotericin B and itraconazole; and (3) correlation of the susceptibility of *Candida albicans* to antifungal agents with the periodontal pocket depth from which the samples had been recovered and to the overall periodontal status of the patients.

## 2. Results

### 2.1. Demographic and Clinical Periodontal Results

The samples were obtained from 163 subjects. Clinical periodontal parameters are presented in Table 1. Oral hygiene index and bleeding score were high (Table 1). A history of antifungal therapy (systemic or local oropharyngeal) was reported by 21 (12.9%) subjects. 

### 2.2. Distribution of Candida Species in Tongue and Subgingival Samples—Primary Outcome

Yeasts of the genus *Candida* were detected in 38/163 (23.3%) of the tongue samples. Among these isolates, the most common species was *C. albicans*, isolated in 29/38 (76.3%) cases, followed by *Candida Glabrata* (15/38; 39.5%), *Candida Tropicalis* (2/38; 5.3%), and *Candida Krusei* (5/38; 13.2%) subjects. Two or more species were isolated from a single tongue sample in 13/38 (34.2%) cases.

*Candida* spp. were found in the subgingival plaque samples from 35/163 (21.5%) subjects. In these samples, *C. albicans* was present in 32/35 cases (91.4%), *Candida Glabrata* in 10/35 cases (28.6%), and *Candida Krusei* in 7/35 (20.0%) samples. In 9/35 (25.7%) of subjects, two or more species were isolated per sample.

**Figure 1:**
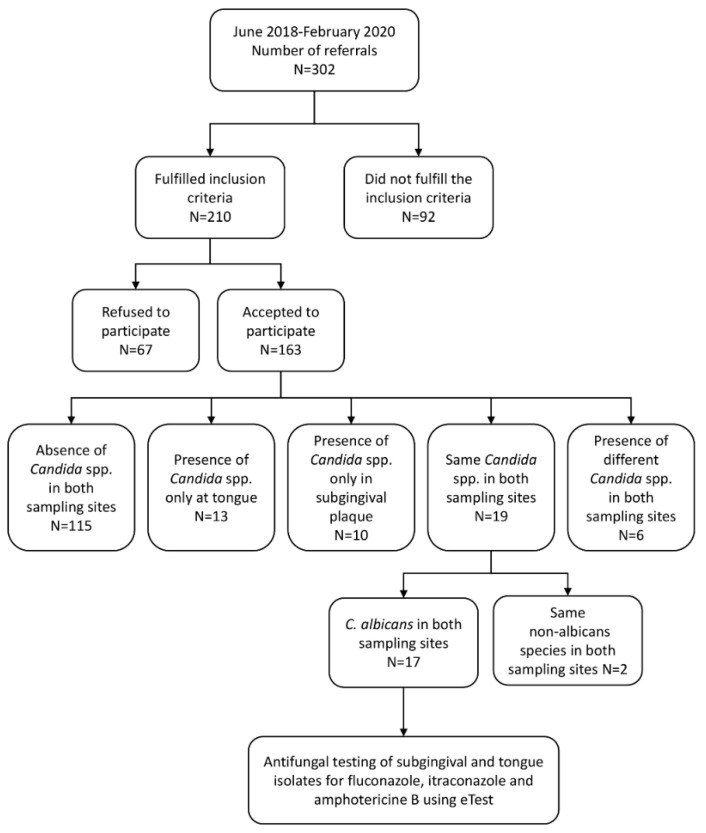
Flow chart of the study participants and study design.

*C. albicans* was detected at both sampling sites in only 17 subjects (Figure 1).

### 2.3. Antifungal Susceptibility Results—Secondary Outcome

Antifungal testing was done on 17 patients in whom *C. albicans* was found in both sampling sites (34 isolates). The Mean Minimal Inhibitory Concentration (MIC) values of fluconazole, amphotericin B and itraconazole were not significantly different between *C. albicans* isolated from the tongue and subgingival biofilm (Table 2). 

MIC values of the present study were interpreted according to the new European Committee on Antimicrobial Susceptibility Testing (EUCAST) breakpoints for antifungal agents’ MIC values, Version 10 [21]. 

The frequency of isolates susceptible to fluconazole was 64.7% and 41.7% for tongue and subgingival plaque, respectively. One subgingival sample (5.87%) showed “I” susceptibility (*χ*^2^, *p* = 0.592). In 15 out of 17 pairs of isolates, the same pattern of susceptibility/resistance was observed. A low frequency of susceptibility to itraconazole was observed for isolates from the tongue (17.6%) as well as from the subgingival areas (11.8%) (*χ*^2^, *p* = 0.628). In 16 out of 17 pairs of isolates, the same pattern of sensitivity/resistance was observed for itraconazole. All 34 isolates were susceptible to amphotericin B.

### 2.4. Relationship between the Susceptibility of Candida albicans to Antifungals and the Clinical Periodontal Parameters

Due to the high number of results—six minimal inhibitory concentration values (three antifungals from two sampling sites) and twelve clinical periodontal parameters—only the statistically significant results (*p* < 0.05) are presented (Table 3). The correlation between full-mouth plaque score (FMPS) and MIC for tongue isolates for all three antimycotics were strongly positive, indicating that MIC increases with the increase of FMPS. Positive correlation was also observed between full-mouth bleeding score (FMBS) and MIC for itraconazole for tongues samples. The same correlation was observed between the percentage of sites 3 mm ≤ clinical attachment loss (CAL) < 5 mm and tongue samples for all three antimycotics and subgingival samples for amphotericin B and fluconazole.

The correlations between probing pocket depth (the deepest pocket of subject) at the sampling site and MIC values were not significant (data is not presented in the table).

### 2.5. Confounding Data Results—Denture Wearers

Five of 17 examined subjects were partial acrylic denture wearers. The mean duration of wearing dentures was 10.4 years; none of them were nocturnal wearers. All subjects maintained denture hygiene using a dental brush and toothpaste only. Nine of ten isolates obtained from these five subjects were resistant to itraconazole and fluconazole.

## 3. Discussion

The prevalence of *Candida* spp. in subgingival areas in healthy subjects is up to 70%, and *C. albicans* has always been the most frequently isolated species [22,23,24]. The incidence of *Candida* spp. in this study was 23.30% in the tongue and 21.50% in subgingival plaque. In our earlier studies, performed on a different study population, the cohorts of healthy subjects with periodontitis showed lower frequency of *Candida* spp. on the tongue, but the presence of subgingival *Candida* spp. varied from 14.3% to even 26.7% [10,25]. In these studies, we concluded that subgingival areas may differ in incidence and/or species distribution of *Candida* from the oral mucosa. We assumed these areas may be a reservoir of microorganisms [10,25]. Subgingival areas and tongue dorsum show diverse ecological properties. Primarily, the subgingival biofilm is attached to a non-shedding hard surface, with different primary colonizers, the redox potential, pH and electrochemical potential and nutrients in the subgingival plaque differing from the tongue as well as the availability of oxygen, giving the subgingival area the potential to develop different biofilms from the tongue [26]. Until recently, the most investigated oral niche of *Candida* sp. was the tongue, followed by buccal and palatal mucosa. Since bacteria were considered as the only etiological factor causing periodontitis until recently, *Candida* was not studied widely in these areas. As a logical extension of our previous work, we continued the investigation with a new research aim—determining the susceptibility of opportunistic pathogens from periodontal pockets (i.e., the reservoirs that are in proximity to systemic circulation) to the effects of commercially available antifungals. 

The mean MIC value for azoles was higher for isolates from periodontal pockets than from the tongue. However, these differences did not reach statistical significance, which may be due to the limited number of tested isolates. Even though both samples were collected from multilayer biofilms from the oral cavity, the different sensitivities of strains from these two sites was expected because of the previously mentioned different conditions, higher complexity and diversity of microorganisms of subgingival biofilms [20]. A strong positive and significant correlation between MICs for isolates from the tongue and full-mouth plaque score (FMPS), as a marker of oral hygiene, was obtained. This means that with a lower level of oral hygiene, which may provide conditions for maturity and higher complexity of biofilms [27], the MIC values increase. In addition, although results did not reach statistical significance, the correlations between MICs for subgingival isolates and full-mouth plaque score were strongly positive, indicating the same relationship. The correlations between moderate periodontal destruction (% 3 mm ≤ clinical attachment loss (CAL) < 5 mm), and MIC were strongly positive and significant. This may lead to an assumption that biofilms from the subjects with moderate destruction provide conditions for yeasts to enhance resistance mechanisms. Conditions within periodontal pockets with moderate destruction, such as serum and nutrient limitation, increased temperature, pH, due to the increase of specific bacteria, and oxygen concentration in the atmosphere around the yeasts’ cells, may influence the yeast virulence and morphologic changes [28,29]. It has been shown that anaerobic conditions may encourage the transition from yeast to hyphal form of *Candida albicans* [30,31]. Additionally, it has been shown that anaerobic conditions inhibit the production of farnesol, which leads to increased resistance of *C. albicans* to antifungals, at least by four-fold [30]. Additionally, *Porphiromonas gingivalis* as key stone periodontal pathogen presented in periodontal pockets with moderate destruction and has mutually protective relationship with *C. albicans* [32]. These bacteria–yeast relationships may, to some extent, explain the significant correlation between moderate periodontal destruction and MIC for *Candida albicans* isolated from subgingival areas. Moreover, Monroy-Perez et al. found that *C. albicans* from moderate periodontitis sites presented higher gene expression of virulence factors and azole resistance with respect to patients with gingivitis or chronic periodontitis. They speculated about the role of these resistant strains in the pathogenesis of periodontitis [22].

On the other hand, regarding itraconazole, susceptible subgingival isolates were related to higher values of mean PPD (at sites higher or equal to 6 mm) than resistant strains. The same relationship was detected when it comes to tongue samples. These confusing relationships may be explained by a complex and dynamic relationship of microorganisms in the subgingival biofilm., For example, the relationship between *C. albicans* and *Fusobacterium nucleatum* as well as *Agregibacter actinomicetemcomitans* has been defined as antagonism [33,34,35]. It should be added that all mentioned bacterial–fungal relations have been proven in vitro, which could be quite different from real subgingival conditions, where a higher number and diversity of microorganisms is present, as well as the influence of the host response. 

The European Committee on Antimicrobial Susceptibility Testing (EUCAST) defines clinical breakpoints for sensitivity for broth microdilution (BMD) susceptibility testing on Roswell Park Memorial Institute (RPMI) 1640 medium. The epsilometer test (E-test) agar gradient MIC method is a commercially available system for antifungal susceptibility, and several studies have explored the reliability of this method, reaching up to 100% [36,37]. Since the E-test presents a modification of the BMD method, and testing in the present study was conducted on RPMI agar, EUCAST clinical breakpoints were applied. In the present study, regarding fluconazole, the frequency of susceptible isolates from the tongue was 64.71%, while this percentage for subgingival area samples was 41.76%. The demonstrated percentage of susceptibility is quite low, especially considering that we tested the strains that represent the reservoirs in subjects without clinical signs of candidiasis. As expected, all tested strains were sensitive to amphotericin B. This result is encouraging, since amphotericin B is the gold standard for the treatment of a wide variety of fungal infections, especially the severe ones. Unlike to azoles, where resistance may occur at several levels (point mutations/upregulation on the gene ERG1, overexpression/mutation on multiple genes for drug efflux pump, etc.) [38], resistance to polyenes is rare. When it occurs, it is due primarily to alterations of membrane sterols [39]. The lowest frequency of sensitivity was detected for itraconazole, where only 11.76% of subgingival strains and 17.64% of strains isolated from the tongue were sensitive. 

It is quite hard to compare the results of our study with the results obtained in similar studies, because we used the latest EUCAST recommendation, and other studies used different and older recommendations. Furlletti et al. applied the Clinical Laboratory Standards Institute (CLSI) breakpoints recommendations. In order to compare our results with those of Furlletti et al., we interpreted them according to the CLSI recommendation (data not presented). We showed a similar pattern of susceptibility and resistance, except for susceptibility to fluconazole for yeasts isolated from subgingival biofilm. Furlletti et al. found a higher percentage of susceptible isolates to fluconazole from subgingival than from oral sites [40]. For itraconazole, we observed a similar percentage of susceptible isolates for tongue and subgingival isolates, similar to Furletti et al. In respect to amphotericin B, the susceptibility of all of our isolated strains to amphotericin B is in accordance with a generally accepted fact that there is a relatively low occurrence of resistance to this antimycotic [41]. Contrary to this, Furlletti et al. found low susceptibility to amphotericin B in both subgingival and oral isolates [40]. It should be stressed that the cited study did not mention the general health of subjects or previous use of antimycotics. Anamnestic data about previous treatments of Candidiasis were collected in our study. Although some of the examined subjects were not sure if they had ever used antifungal topical therapy, they were quite sure about systemic antifungal treatment, which means that they were probably not treated with amphotericin B. This fact and the small number of isolates may be the reason for 100% susceptibility of isolates to amphotericin B. Kaminska et al. examined oral specimens isolated from children’s (0–18 y) buccal swabs to multiple antifungals, also using commercially available tests [42]. Their breakpoints, defined by themselves, were also different from the latest EUCAST recommendation. When our results are interpreted based on these breakpoints, they are concordant with the results of Kaminska et al. [42]. However, the mentioned studies were conducted on Brazilian, Mexican and Polish populations.

Similar studies have not been performed for this geographic region. The epidemiology of susceptibility to antimicrobial agents of microorganisms is geographically specific and is, therefore, important and critical when choosing the empiric therapy [43]. In cases of systemic and severe fungal infections, diagnostic procedures are time consuming, and empiric therapy is vital. Since subgingival biofilms are reservoirs of microorganisms, determining the susceptibility of yeasts originating from these biofilms is important. Although antifungal testing was not performed on biofilms, the tested isolates were sampled from biofilms. It has been proven that resuspended yeasts, detached from the biofilm, were more resistant to amphotericin B than planktonic cells, although less resistant than biofilm cells [44]. To the best of our knowledge, the only reported study from Serbia on the susceptibility of *Candida* spp. isolates to antifungal medication was that of Jelesić et al.; they tested isolates from different sites (blood and feces) from subjects with clinical signs of disease [45]. All *C. albicans* isolates were susceptible to amphotericin B, while 96% were susceptible to all examined agents—amphotericin B, fluconazole, itraconazole and voriconazole. 

In everyday clinical practice, antimycotics, primary azoles and polyenes are frequently used for the treatment of oral candidiasis. There are some concerning facts about antifungal treatment. First, antifungals are often unnecessarily used in the treatment of oral conditions clinically misdiagnosed as *Candida* infections (e.g., geographic tongue [46], burning mouth syndrome, oral allergies, etc.). Second, antifungal therapy is used whenever *Candida* spp. is isolated from oral samples, overlooking the fact that *Candida* sp. is considered a commensal; thus, microbial results should be interpreted with caution [47]. Third, some cases of refractory candidiasis have an underlying systemic condition (e.g., anemia, diabetes, high-carbohydrate diet, etc.). In these cases, the infection is treated repeatedly by antifungals, without treating the underlying condition. Sometimes, the underlying conditions for oral candidiasis may simply be the presence of acrylic dentures [48]. In this study, 5 out of 17 subjects were denture wearers. Although none of them were nocturnal wearers and all were systemic healthy, 9 out of 10 isolates were not susceptible to both azoles tested. It has been shown that denture wearing may change the phenotype of *Candida albicans* in the sense of increasing its virulence [49]. Although isolates from denture surfaces are more resistant to antimycotics [50], there are also studies that show no impact of denture wearing on the antifungal susceptibility of *Candida* isolated from the tongue [51]. To avoid the bias as a consequence of difference in the susceptibility of different *Candida* species, we performed these tests only where the same species was isolated from both sites. This brought us to a small number of isolates, which is the main limitation of this study. These results should encourage other in vitro and in vivo studies in order to define empiric antifungal therapy. Further analysis comprising a higher number of subjects and antifungal testing for different *Candida* spp. is needed. 

## 4. Materials and Methods

### 4.1. Study Design, Ethical Approval and Inclusion Criteria

This single-center, cross-sectional observational study was conducted between June 2018 and February 2020. The subjects were referred for the treatment of periodontitis to the Department of Oral Medicine and Periodontology, School of Dental Medicine, University of Belgrade. 

The study was approved by the Ethical Committee of the School of Dental Medicine, University of Belgrade (No 36/14), in accordance with the Declaration of Helsinki. Every subject was informed about the study and signed an informed consent before entering the study. 

This survey followed the Strengthening the Reporting of Observational studies in Epidemiology (STROBE) guidelines [52]. The number of screened participants is shown in the flow diagram (Figure 1). Participants were collected according to inclusion criteria. Based on the results of the study, the post hoc achieved power was 90%. The power for 17 respondents was calculated for difference between two independent means, α = 0.05; effect size was calculated based on mean and SD of difference between two independent means. This was performed in Gpower program (version 3.1.9.2, Heinrich-Heine-Universität Düsseldorf, Düsseldorf, Germany).

Data on the past treatment of candidiasis, either systemic or local, were obtained. The presence of dentures, as well as information about the duration of its usage and nocturnal wearing habits, were recorded.

Inclusion criteria were absence of any symptoms or clinical signs of candidiasis (acute or chronic), presence of periodontitis and no history of periodontal treatment. 

Exclusion criteria were age <18 or >80 years, edentulism, presence of chronic systemic diseases and consumption of antibiotics less than 1 month prior to the exam.

Patients who fulfilled the inclusion/exclusion criteria and agreed to participate in the study were included.

The samples were collected from 163 subjects with periodontitis. Finally, after microbiological diagnoses and analyses, the isolates obtained from 17 patients (2 isolates per subject) were eligible for antimicrobial testing (detailed explanation is provided later in the manuscript).

### 4.2. Periodontal Assessment and Diagnosis of Periodontitis

Hard and soft oral tissues (lips, buccal mucosa and tongue, hard and soft palate) were examined by inspection.

Periodontitis was defined by the criteria of the World Workshop on the Classification of Periodontal and Peri-implant Diseases and Conditions [53].

A full-mouth clinical periodontal examination was performed by one calibrated periodontist, using a manual periodontal probe graduated in millimeters (PCPUNC-15; HU-Friedy, Chicago, IL, USA). The examiner was considered calibrated when the percentage of agreement within 1.0 mm between two measurements, obtained in duplicate from 10 patients outside the study, was ≥90%, with a Kappa coefficient ≥0.7 [54].

The following clinical parameters were measured at six sites per each tooth (third molars were excluded): plaque index (PI) [55]; bleeding on probing (BOP); probing pocket depth (PPD); clinical attachment level (CAL). PPD and CAL were expressed in mm and rounded to the nearest millimeter. 

Using the mentioned measurements, the following parameters were calculated:

(1) full-mouth plaque score (FMPS)—expressed as the percentage of sites with soft or mineral debris;
(2) full-mouth bleeding score (FMBS)—expressed as the percentage of bleeding sites 15 s after probing;
(3) mean PPD—mean value for full-mouth probing pocket depth;
(4) mean probing pocket depth at sites with PPD ≥ 6 mm;
(5) mean CAL—mean value for full-mouth clinical attachment loss;
(6) mean CAL at sites ≥ 5 mm;
(7) % of sites 4 mm ≤ PPD < 6 mm—calculated as the percentage of sites with a probing depth of 4 or 5 mm relative to all measured sites;
(8) % of sites PPD ≥6 mm—calculated as the percentage of sites with a probing depth of 6 mm or deeper relative to all measured sites;
(9) No. of sites PPD ≥6 mm—the number of sites with a probing pocket depth of 6 mm or more;
(10) % of sites 3 mm ≤ CAL < 5 mm—calculated as the percentage of sites with clinical attachment loss of 3 or 4 mm relative to all measured sites;
(11) % of sites CAL ≥ 5 mm—calculated as the percentage of sites with clinical attachment loss of 5 mm or higher relative to all measured sites.

### 4.3. Microbiological Sampling Procedures and Analysis

The sampling procedures were performed one day after clinical examination on 163 patients included in the study.

Oral swab specimens were collected by swabbing ten times from the dorsum of the tongue using a sterile cotton stick. The swabs were immediately inoculated on Sabouraud dextrose agar (SDA, Oxoid, Basingstoke, UK). The subgingival samples were collected from the deepest probing pocket depth site. The selected tooth was first isolated by cotton rolls and air-dried. Then, supragingival plaques were gently removed by curettes and sterile gauze. Subgingival biofilm was obtained using a sterile curette (Mini Five, Hu-Friedy, Chicago, IL, USA). A curette was inserted into the bottom of the deepest pocket, and subgingival plaque was sampled by pulling the sharp edge of the curette against the tooth with an upward motion. The subgingival samples were inoculated in sterile plastic tubes containing 1 mL of Sabouraud dextrose broth. These tubes were vortexed for 60 s, and 20 μL of broth was streaked on Sabouraud dextrose agar in duplicate using a sterile plastic micro pipette. The tongue and subgingival samples were incubated at 37 °C for 48 h in aerobic conditions. CHROMagar Candida Medium (Becton Dickinson, Heidelberg, Germany), the germ-tube production test and carbohydrate assimilation test were used for the distinction of different *Candida* species—*Candida albicans* (*C. albicans*), *C. dubliniensis*, *C. glabrata*, *C. tropicalis* and *C. krusei* [56].

### 4.4. Antifungal Susceptibility Testing

The final selection of samples for antifungal testing was carried out in order to avoid the bias of difference in the susceptibility to antimycotics caused by the species (e.g., higher resistance of *C. glabrata* to fluconazole) or individual subjects’ characteristics (e.g., a history of usage of antifungals). These resulted in antifungal susceptibility testing of the samples (N = 34) obtained from the subjects (N = 17), with the same *Candida* species isolated from both sampling sites (the tongue and subgingival area). 

The fungal suspensions of these 34 isolates were prepared so that the turbidity corresponded to 0.5 McFarland standard (≈10^6^ cells/mL) by scraping yeasts with a sterile loop and mixing them with sterile saline. The turbidity was verified by a densitometer (DEN-1 densitometer, Biosan, Latvia). The suspensions were inoculated on RPMI agar medium (Biomerieux, Basingstoke, UK) by swabbing in three directions. Antimicrobial testing was done using a commercially available E-test (Liofilchem^®^ MIC Test Strip, Roseto degli Abruzzi TE, Italy) for fluconazole, amphotericin B and itraconazole. A sterile E-test strip was placed in the middle of the medium and incubated for 20 h at 37 °C, according to the manufacturer’s instructions. For amphotericin B, the MIC was read as the concentration on the strip where the complete inhibition of growth was detected. The lowest concentration at which the border of the elliptical inhibition zone decreases in growth (visually 80% of inhibition), interpreted from the scale on the strip, was read as the MIC for azoles: itraconazole and fluconazole. Numerically, the results for each strain were obtained as the concentration range presented at the E-test strip: 0.016–256 μL/mL for fluconazole and 0.002–32 μL/mL for both amphotericin B and itraconazole. The obtained MIC values were compared to the interpretative criteria of the European Committee on Antimicrobial Susceptibility Testing 2020 (EUCAST) [21]. These recommendations define isolates as Susceptible, standard dosing regimen (S), Resistant (R), and Susceptible, increased exposure (I) if the MIC values are between R and S breakpoints. 

### 4.5. Study Outcomes

The primary outcome was the determination of the frequencies of *Candida* spp. on the tongue and in periodontal pocket biofilms. The secondary outcomes were antifungal susceptibility results—the MIC values and the susceptibility as defined according to the EUCAST 2020 breakpoints. Additionally, analyses of the relationship between antifungal susceptibility and periodontal parameters were performed.

### 4.6. Statistical Analysis

The SPSS 22.0 software package for Windows (SPSS Inc., Chicago, IL, USA) was used for statistical analysis. Descriptive data were presented as Mean ± SD (min–max) for numerical and percentages for categorical variables. Normality of the numeric data was tested by the Koglomorov–Smirnov Test. Student’s *t*-test and One-Way ANOVA was used for normally distributed data. Parametric data were analyzed using the Mann–Whitney test or Kruskall–Wallis Test. The Chi Square (*χ*^2^) Test was used for comparison of categorical variables. Spearman’s correlation coefficient was calculated in order to assess the relationship between the MICs for antifungals and clinical periodontal parameters. Differences were considered significant when the *p*-value was < 0.05.

## 5. Conclusions

This study observed that the presence of *Candida* spp. on tongue samples differed from subgingival samples of periodontitis patients without candidiasis, both in frequency of isolation and findings of different species. The same species found in both sampling sites were found only in 19/163 patients, out of which 17 were *Candida albicans*. 

A rather low frequency of susceptibility to fluconazole and itraconazole of *Candida albicans* isolates both from the tongue (64.7% and 17.6%, respectively) and subgingival sites (41.7% and 11.8%, respectively) was observed. All isolates from both sites are susceptible to amphotericin B. Although subgingival isolates seemed to show higher resistance to fluconazole and itraconazole; due to the limited number of isolates, these differences did not show statistical significance. 

The highest resistance to fluconazole and itraconazole was found in subjects with the lowest hygiene level (highest FMPS) and moderate periodontal destruction.

## Figures and Tables

**Table 1:** Clinical periodontal parameters of subjects.

Variable Mean ± SD (Min–Max)	
No. of teeth	20.88 ± 4.90
FMPS (%)	72.75 ± 26.74
FMBS (%)	62.07 ± 27.71
Mean PPD (mm)	3.81 ± 1.06
Mean CAL (mm)	4.04 ± 1.59
Mean PPD at sites ≥6 mm (mm)	6.69 ± 0.88
Mean CAL at sites ≥5 mm (mm)	6.14 ± 1.06
% of sites 4 mm ≤ PPD < 6 mm	27.65 ± 15.79
% of sites PPD ≥ 6 mm	11.45 ± 18.06
No. of sites PPD ≥ 6 mm	14.84 ± 23.67
% of sites 3 mm ≤ CAL < 5 mm	32.36 ± 16.04
% of sites CAL ≥ 5 mm	29.35 ± 26.98

SD—standard deviation; FMPS—full-mouth plaque score; FMBS—full-mouth bleeding score; PPD—probing pocket depth; CAL—clinical attachment loss.

**Table 2:** Minimal inhibitory concentrations of Fluconazole, Amphotericin B and Itraconazole for *Candida albicans* isolated from the tongue and from subgingival biofilm.

		Tongue Sample	Subgingival Sample	*p* Value
		Mean ± SD Median (Min–Max)	Mean ± SD Median (Min–Max)	
MIC (μL/mL)	Fluconazole	4.79 ± 4.61 2.00 (0.38–16.00)	5.88 ± 8.13 2.00 (1.00–32.00)	0.709
	Amphotericin B	0.42 ± 0.27 0.25 (0.19–1.00)	0.38 ± 0.18 0.25 (0.19–0.75)	0.919
	Itraconazole	2.22 ± 7.69 0.12 (0.019–32)	2.80 ± 7.78 0.12 (0.019–32)	0.708

Statistical analysis was performed using Mann-Whitney U Test.

**Table 3:** Correlations between MIC values of tested antifungals and clinical periodontal parameters.

Clinical Parameter	Antimycotic (MIC)	Sampling Site	R	*p* Value
FMPS	Amphotericin B	Tongue	0.566	0.018
	Fluconazole		0.681	0.003
	Itraconazole		0.529	0.029
FMBS	Itraconazole	Tongue	0.524	0.031
Mean PPD at sites ≥ 6mm	Itraconazole	Tongue	−0.723	0.012
% site 3 mm ≤ CAL < 5 mm	Amphotericin B	Tongue	0.596	0.012
	Amphotericin	Periodontal pocket	0.557	0.020
	Fluconazole	Tongue	0.639	0.006
	Fluconazole	Periodontal pocket	0.592	0.012
	Itraconazole	Tongue	0.547	0.023

R—Spearman correlation coefficient. Note: The correlation between six MIC values (three antifungals for both sampling sites) and clinical periodontal parameters (FMPS, FMBS, full-mouth PPD, mean PPD at sites ≥ 6 mm, full-mouth CAL, mean CAL for sites ≥ 5 mm, % sites 4 mm ≤ PPD < 6mm, % sites PPD ≥ 6 mm, % sites 3 mm ≤ CAL < 5 mm, % sites CAL ≥ 5 mm) were calculated. Table 3 presents only statistically the significant correlations due to the high number of obtained data.

## Data Availability

The data presented in this study are available on request from the corresponding authors. The data are not publicly available because this is the first part of ongoing research.

## References

[1] Arweiler N.B., Netuschil L. (2016). The oral microbiota. Microbiota Hum. Body.

[2] Colombo A.P.V., Magalhães C.B., Hartenbach F.A.R.R., do Souto R.M., da Silva-Boghossian C.M. (2016). Periodontal-disease-associated biofilm: A reservoir for pathogens of medical importance. Microb. Pathog..

[3] Meto A., Colombari B., Sala A., Pericolini E., Meto A., Peppoloni S., Blasi E. (2019). Antimicrobial and antibiofilm efficacy of a copper/calcium hydroxide-based endodontic paste against *Staphylococcus aureus*, *Pseudomonas aeruginosa* and *Candida albicans*. Dent. Mater. J..

[4] Alberti A., Corbella S., Taschieri S., Francetti L., Fakhruddin K.S., Samaranayake L.P. (2021). Fungal species in endodontic infections: A systematic review and meta-analysis. PLoS ONE.

[5] Akpan A., Morgan R. (2002). Oral candidiasis. Postgrad. Med. J..

[6] Bertolini M., Dongari-Bagtzoglou A. (2019). The relationship of *Candida albicans* with the oral bacterial microbiome in health and disease. Oral Mucosal Immunity and Microbiome.

[7] Krüger W., Vielreicher S., Kapitan M., Jacobsen I.D., Niemiec M.J. (2019). Fungal-bacterial interactions in health and disease. Pathogens.

[8] Boyer E., Martin B., Le Gall-David S., Fong S.B., Deugnier Y., Bonnaure-Mallet M., Meuric V. (2020). Periodontal pathogens and clinical parameters in chronic periodontitis. Mol. Oral Microbiol..

[9] Klimesova K., Jiraskova Zakostelska Z., Tlaskalova-Hogenova H. (2018). Oral bacterial and fungal microbiome impacts colorectal carcinogenesis. Front. Microbiol..

[10] Matic Petrovic S., Radunovic M., Barac M., Kuzmanovic Pficer J., Pavlica D., Arsic Arsenijevic V., Pucar A. (2019). Subgingival areas as potential reservoirs of different *Candida* spp in type 2 diabetes patients and healthy subjects. PLoS ONE.

[11] Han Y.W., Wang X. (2013). Mobile microbiome: Oral bacteria in extra-oral infections and inflammation. J. Dent. Res..

[12] McCarty T.P., Pappas P.G. (2016). Invasive candidiasis. Infect. Dis. Clin..

[13] Terzic M., Aimagambetova G., Terzic S., Radunovic M., Bapayeva G., Laganà A.S. (2021). Periodontal pathogens and preterm birth: Current knowledge and further interventions. Pathogens.

[14] Sanz M., Marco del Castillo A., Jepsen S., Gonzalez-Juanatey J.R., D’Aiuto F., Bouchard P., Chapple I., Dietrich T., Gotsman I., Graziani F. (2020). Periodontitis and cardiovascular diseases: Consensus report. J. Clin. Periodontol..

[15] Liccardo D., Cannavo A., Spagnuolo G., Ferrara N., Cittadini A., Rengo C., Rengo G. (2019). Periodontal Disease: A Risk Factor for Diabetes and Cardiovascular Disease. Int. J. Mol. Sci..

[16] Jeronimo L.S., Abreu L.G., Cunha F.A., Lima R.E. (2020). Association between periodontitis and nosocomial pneumonia: A systematic review and meta-analysis of observational studies. Oral Health Prev. Dent..

[17] Gülses A., Açil Y., Wiltfang J. (2020). Oral surgery related fungal endocarditis: The need for a novel concept in endocarditis prophylaxy. Med. Hypotheses.

[18] Sedlacek M., Walker C. (2007). Antibiotic resistance in an in vitro subgingival biofilm model. Oral Microbiol. Immunol..

[19] Al-Fattani M.A., Douglas L.J. (2004). Penetration of Candida biofilms by antifungal agents. Antimicrob. Agents Chemother..

[20] Kim S.-M., Kim H.C., Lee S.-W.S. (2011). Characterization of antibiotic resistance determinants in oral biofilms. J. Microbiol..

[21] Breakpoint Tables for Interpretation of MICs and Zone Diameters, Version 10.0, 2020. CLinical Breakpoint for Fungi. https://www.eucast.org/astoffungi/clinicalbreakpointsforantifungals.

[22] Monroy-Pérez E., Rodríguez-Bedolla R.M., Garzón J., Vaca-Paniagua F., Jiménez E.A.-R., Paniagua-Contreras G.L. (2020). Marked virulence and azole resistance in *Candida albicans* isolated from patients with periodontal disease. Microb. Pathog..

[23] Krishnan G.S., Naik D., Uppoor A., Nayak S., Baliga S., Maddi A. (2020). Candidal carriage in saliva and subgingival plaque among smokers and non-smokers with chronic periodontitis—A cross-sectional study. PeerJ.

[24] Urzúa B., Hermosilla G., Gamonal J., Morales-Bozo I., Canals M., Barahona S., Cóccola C., Cifuentes V. (2008). Yeast diversity in the oral microbiota of subjects with periodontitis: *Candida albicans* and *Candida dubliniensis* colonize the periodontal pockets. Sabouraudia.

[25] Cimbaljević M., Radunović M., Jotić A., Pucar A. (2015). Detection and sampling methods for isolation of *Candida* spp. from oral cavities in diabetics and non-diabetics. Braz. Oral Res..

[26] Marsh P.D. (2000). Role of the Oral Microflora in Health. Microb. Ecol. Health Dis..

[27] Thomas J.G., Nakaishi L.A. (2006). Managing the complexity of a dynamic biofilm. J. Am. Dent. Assoc..

[28] Rosa E.A.R., Rached R.N., Ignácio S.A., Rosa R.T., da Silva W.J., Yau J.Y.Y., Samaranayake L.P. (2008). Phenotypic evaluation of the effect of anaerobiosis on some virulence attributes of *Candida albicans*. J. Med. Microbiol..

[29] Shapiro R.S., Ryan O., Boone C., Cowen L.E. (2012). Regulatory circuitry governing morphogenesis in *Saccharomyces cerevisiae* and *Candida albicans*. Cell Cycle.

[30] Dumitru R., Hornby J.M., Nickerson K.W. (2004). Defined anaerobic growth medium for studying *Candida albicans* basic biology and resistance to eight antifungal drugs. Antimicrob. Agents Chemother..

[31] Biswas S.K., Chaffin W.L. (2005). Anaerobic growth of *Candida albicans* does not support biofilm formation under similar conditions used for aerobic biofilm. Curr. Microbiol..

[32] Karkowska-Kuleta J., Bartnicka D., Zawrotniak M., Zielinska G., Kierońska A., Bochenska O., Ciaston I., Koziel J., Potempa J., Baster Z. (2018). The activity of bacterial peptidylarginine deiminase is important during formation of dual-species biofilm by periodontal pathogen *Porphyromonas gingivalis* and opportunistic fungus *Candida albicans*. Pathog. Dis..

[33] Bachtiar E.W., Bachtiar B.M., Jarosz L.M., Amir L.R., Sunarto H., Ganin H., Meijler M.M., Krom B.P. (2014). AI-2 of *Aggregatibacter actinomycetemcomitans* inhibits *Candida albicans* biofilm formation. Front. Cell. Infect. Microbiol..

[34] Bor B., Cen L., Agnello M., Shi W., He X. (2016). Morphological and physiological changes induced by contact-dependent interaction between *Candida albicans* and *Fusobacterium nucleatum*. Sci. Rep..

[35] Bachtiar E.W., Bachtiar B.M. (2020). Effect of cell-free spent media prepared from *Aggregatibacter actinomycetemcomitans* on the growth of *Candida albicans* and *Streptococcus mutans* in co-species biofilms. Eur. J. Oral Sci..

[36] Arendrup M., Lundgren B., Jensen I.M., Hansen B.S., Frimodt-Møller N. (2001). Comparison of Etest and a tablet diffusion test with the NCCLS broth microdilution method for fluconazole and amphotericin B susceptibility testing of Candida isolates. J. Antimicrob. Chemother..

[37] Elder J.V. (1996). Fluconazole and amphotericine B antifungal susceptibility testing by National Committee for Clinical Laboratory Standards broth macrodilution method compared with E test and semiautomated broth microdilution test. J. Clin. Microbiol..

[38] Sharma J., Rosiana S., Razzaq I., Shapiro R.S. (2019). Linking Cellular Morphogenesis with Antifungal Treatment and Susceptibility in Candida Pathogens. J. Fungi.

[39] Jensen R.H., Astvad K.M.T., Silva L.V., Sanglard D., Jørgensen R., Nielsen K.F., Mathiasen E.G., Doroudian G., Perlin D.S., Arendrup M.C. (2015). Stepwise emergence of azole, echinocandin and amphotericin B multidrug resistance in vivo in *Candida albicans* orchestrated by multiple genetic alterations. J. Antimicrob. Chemother..

[40] Furlletti V.F., de Cássia Mardegan R., Obando-Pereda G.A., Aníbal P.C., Duarte M.C.T., Gonçalves R.B., Höfling J.F. (2008). Susceptibility of Candida spp. Oral isolates for azolic antifungals and amphotericin B. Braz. J. Oral Sci..

[41] Ellis D. (2002). Amphotericin B: Spectrum and resistance. J. Antimicrob. Chemother..

[42] Kamińska A., Malm A., Szymańska J. (2019). Antifungal drugs resistance profiles of *C. albicans* strains isolated from the oral cavity of children and adolescents. Acta Pol. Pharm. Drug Res..

[43] Pfaller M.A. (2012). Antifungal drug resistance: Mechanisms, epidemiology, and consequences for treatment. Am. J. Med..

[44] Baillie G.S., Douglas L.J. (1998). Effect of growth rate on resistance of *Candida albicans* biofilms to antifungal agents. Antimicrob. Agents Chemother..

[45] Jelesić Z.Z., Medić D.D., Mihajlović-Ukropina M.M., Jevtić M., Gusman V.P., Radosavljević B.J., Milosavljević B.T. (2011). Susceptibility to antifungal agents of *Candida* spp. from blood and feces collected in Novi Sad in 3-year period (2008–2010). Zb. Matice Srp. Za Prir. Nauk..

[46] Matić-Petrović S., Đorđević M., Radunović M., Živanović T., Pavlica D., Pucar A. (2019). Geographic tongue: Does Candida play a role in its pathogenesis. Balk. J. Dent. Med..

[47] Arendrup M.C. (2013). Candida and candidaemia. *Susceptibility and epidemiology*. Dan. Med. J..

[48] Volpato Sanitá P., Lúcia Machado A., Cláudia Pavarina A., Maria Sgavioli Massucato E., Lopes Colombo A., Eduardo Vergani C. (2012). Microwave denture disinfection versus nystatin in treating patients with well-controlled type 2 diabetes and denture stomatitis: A randomized clinical trial. Int. J. Prosthodont..

[49] Bachtiar B.M., Fath T., Widowati R., Bachtiar E.W. (2020). Quantification and pathogenicity of *Candida albicans* in denture-wearing and nondenture-wearing elderly. Eur. J. Dent..

[50] Chandra J., Mukherjee P., Leidich S., Faddoul F., Hoyer L., Douglas L., Ghannoum M. (2001). Antifungal resistance of candidal biofilms formed on denture acrylic in vitro. J. Dent. Res..

[51] Lyon J., Moreira L., Cardoso M., Saade J., Resende M. (2008). Antifungal suscepitibility profile of *Candida* spp. oral isolates obtained from denture wearers. Braz. J. Microbiol..

[52] Vandenbroucke J.P., von Elm E., Altman D.G., Gøtzsche P.C., Mulrow C.D., Pocock S.J., Poole C., Schlesselman J.J., Egger M. (2014). Strengthening the Reporting of Observational Studies in Epidemiology (STROBE): Explanation and elaboration. Int. J. Surg..

[53] Caton J.G., Armitage G., Berglundh T., Chapple I.L., Jepsen S., Kornman K.S., Mealey B.L., Papapanou P.N., Sanz M., Tonetti M.S. (2018). A new classification scheme for periodontal and peri-implant diseases and conditions–Introduction and key changes from the 1999 classification. J. Periodontol..

[54] Vargas-Villafuerte K.R., Dantas F.T., Messora M.R., Novaes A.B., Grisi M.F., Taba Jr M., Souza S.L., Candido dos Reis F.J., Carrara H.H., Palioto D.B. (2016). Preliminary results of non-surgical periodontal treatment in patients with breast cancer undergoing chemotherapy. J. Periodontol..

[55] O'leary T.J. (1972). The plaque control record. J. Periodontol..

[56] Marinho S.A., Teixeira A.B., Santos O.S., Cazanova R.F., Ferreira C.A.S., Cherubini K., Oliveira S.D.d. (2010). Identification of *Candida* spp. by phenotypic tests and PCR. Braz. J. Microbiol..

